# *Prosorhynchus crucibulum* (Digenea: Bucephalidae) miracidium morphology and its passive transmission pattern

**DOI:** 10.1051/parasite/2012193277

**Published:** 2012-08-15

**Authors:** C.J. Francisco, M.A. Hermida, M.J. Santos

**Affiliations:** 1 Universidade do Porto, Faculdade de Ciências, Departamento de Biologia, Rua do Campo Alegre, s/n., FC4, 4169-007 Porto, Portugal, and CIMAR Laboratório Associado / CIIMAR, Centro de Investigação Marinha e Ambiental Rua dos Bragas, 289 4050-123 Porto Portugal

**Keywords:** *Prosorhynchus crucibulum*, egg, miracidia, transmission, morphology, *Prosorhynchus crucibulum*, oeuf, miracidium, transmission, morphologie

## Abstract

The characterization of *Prosorhynchus crucibulum* (Rudolphi, 1819) Odhner, 1905 egg and miracidium is important in order to better understand the transmission dynamics between the definitive host and the primary host, the mussel. In this way, the objective of this work was to study the miracidia morphology, in order to evaluate if this species belongs to the group of passive or active transmission larvae. The morphology of eggs is similar to the ones presented by other *Prosorhynchus* species, with a small size of 26 × 17 μm, and four-five times smaller than the ones of *Fasciola hepatica*. The number of eggs produced per worm was around 6,760 (4,236-8,401), which was four-five times higher than in *F. hepatica*. The miracidia presented small dimensions 24 *×* 15 μm (23-25 × 13-15 μm range), a long stylet, two ciliated epithelial plates, very long cilia (12.7 μm) and absence of terebratorium and eyespots. Those features of the miracidia suggest that *P. crucibulum* belongs to the group of passively infecting larvae.

## Abstract (fr)

La morphologie des oeufs et du miracidium de *Prosorhynchus crucibulum* (Rudolphi, 1819) Odhner, 1905 est importante afin de mieux comprendre le mode de transmission entre l’hôte définitif et l’hôte intermédiaire, la moule. L’objectif de ce travail était d’étudier la morphologie du miracidium afin de savoir si l’espèce appartient au groupe des larves à transmission passive ou active. La morphologie des oeufs est semblable à celle d’autres espèces de *Prosorhynchus*, avec une taille de 26 × 17 μm, quatre-cinq fois plus petite que celle de *Fasciola hepatica*. Le nombre moyen d’oeufs produits par ver est de 6 760 (4 236-8 401), quatre-cinq fois plus élevé que celui observé avec *F. hepatica*. Le miracidium est également de petite dimension, 24 × 15 μm (23-25 × 13-15 μm), avec un long stylet, les deux plans épithéliaux ciliés, des cils de dimension très longue (12,7 μm) et l’absence de terebratorium et d’ocelle. Ces caractéristiques du miracidium de *P. crucibulum* suggèrent que l’espèce appartient au groupe des larves à infection passive.

The mussel (*Mytilus* spp.), a highly appreciated mollusc and therefore an important commercial species in southern Europe, is the first intermediate host of the bucephalid digenean *Prosorhynchus crucibulum* (Rudolphi, 1819) Odhner, 1905 (Matthews 1973, Cousteau *et al.*, 1990; McGladdery *et al.*, 1999). Moreover, *Prosorhynchus* sp. infection had been described as causing serious problems in mussel, like castration and weakening of the adductor muscle (Cousteau *et al.*, 1990; Cousteau *et al.*, 1993; Shelley *et al.*, 1988; Lasiak, 1992; Calvo & Mcquaid, 1998; Silva *et al.*, 2002; Cochôa & Magalhães, 2008; Francisco *et al.*, 2010). Although there are some studies on the ecology, biology and morphology characteristics of adult and metacercariae of the genus *Prosorhynchus* Odhner, 1905 (Jones, 1943; Matthews, 1973; Santos & Gibson, 2002; Laffargue *et al.*, 2004; Etchegoin *et al.*, 2005; Bray & Justine, 2006), information about the egg or miracidium stages and it life cycle dynamics are scantily presented. Recently, the miracidium active way of infection in Bucephalidae was questioned (Galaktionov & Dobrovolskij, 2003). Therefore, the main aim of this work was to characterize the transmission of egg and/or miracidium *P. crucibulum* to the first host, and characterize the larva morphology comparing with *Fasciola hepatica* in order to classify them as passive or active miracidium.

## Material and Methods

Adult worms of *P. crucibulum* (n = 14) were collected from four freshly caught conger eels (*Conger conger*), its definitive host. First, we used *P. crucibulum* (n = 8), to estimate average number of eggs per adult worm. The number of eggs per adult from *F. hepatica* (n = 2) was also counted for comparison purposes.

**Fig. 1.**
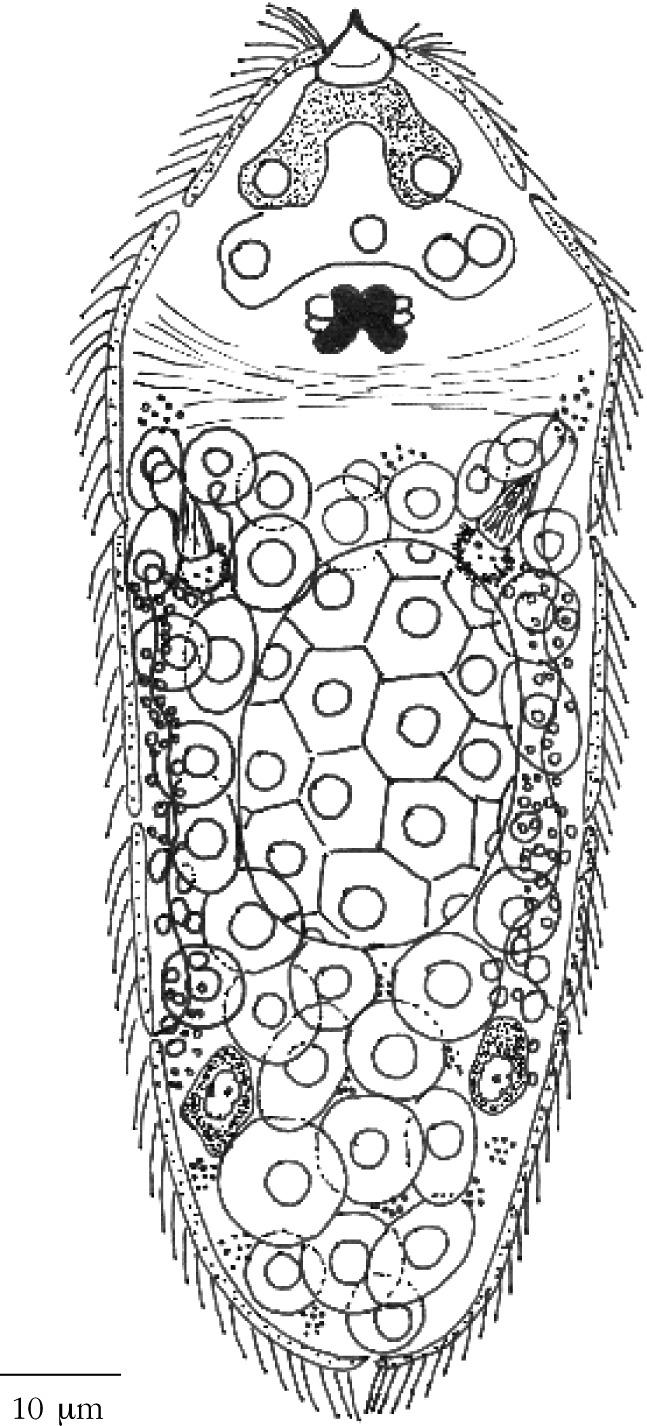
Miracidium of *Fasciola hepatica* (Linnaeus, 1758) redrawn and adapted from Kearn (1997).

The study of *P. crucibulum* egg and miracidium morphology was performed by light microscopy (LM), and the observations were made with a Zeiss Axiophot microscope, equipped with a digital camera Zeiss Axiocam Icc3 and image analysis software (AxioVision 4.6). The eggs were placed in a small drop of saline water (35 ‰ salinity) on a slide and analysed. The live miracidia morphology was studied from eggs that were artificially hatched, by pressing them with a cover glass; some were observed fresh while others were later stained in methylene blue or eosin. The miracidium morphology of *F. hepatica*, an active infective larvae, was redrawn here for comparison purposes (Fig. 1) with our species.

## Results

The estimated average number of eggs per adult worm (n = 8) in *P. crucibulum* was 6,760 (4,236-8,401). The percentage and average number ± standard deviation (range) of immature and mature eggs in the adult worms was 21%, 1,396 (281- 2,233) and 79%, 5,363 (3,331-7,278), respectively. For comparison with our values we determined the same variables in *F. hepatica*. The minimum number of eggs (n = 2) in adult worms of *F. hepatica* and its average number was 1,459 ± 730 (296-1,163), presenting a ratio of young and mature eggs of 16% [114 ± 59 (72-156)] and 84% [616 ± 554 (224-1,007)].

The eggs from *P. crucibulum* (n = 10) presented 26 × 17 µm in average size and 24-27 × 11-20 µm in range (Table I). The shell coloration of the eggs varied between transparent for the immature eggs and greenchestnut for the mature eggs.

**Table I.** Measurements of *Prosorhynchus* spp. eggs.

*Prosorhynchus* species	Definitive host	Locality	n	Egg dimensions (µm)	References
*P. crucibulum*	*Conger conger*	Portugal	8	26 × 17	Present study
*P. aculeatus*	*C. conger*	Great Britain	–	27 × 18	Jones (1943)
*P. maternus*	*Epinefilus malabaricus*	New Caledonia	8	28 × 19	Bray & Justine (2006)
*P. pacificus*	*E. analogus*	Mexico	5	29–33 × 19–20	Winter (1960)
*P. pacificus*	*E. tauvina*	Bay of Bengal	3	32 × 19	Bray & Justine (2006)
*P. pacificus*	*Mycteroperca olfax*	Galapagos	2	28–31 × 15–16	Bray & Justine (2006)
*P. atlanticus*	*M. bonaci*	Florida	3	34 × 19	Bray & Justine (2006)
*P. australis*	*C. orbignianus*	Argentina	14	32 × 19	Bray & Justine (2006)

**Fig. 2.**
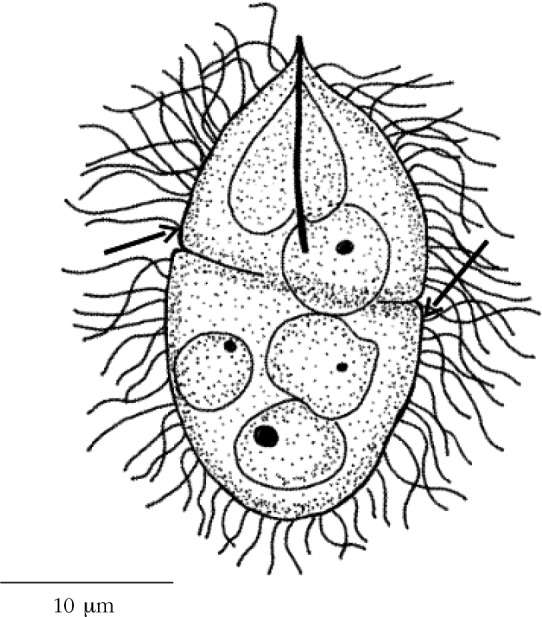
Drawing of a miracidium of *Prosorhynchus crucibulum* (Rudolphi, 1819) Odner, 1905, observed with light microscopy, covered with peripheral cilia and showing two epithelial plates (arrows) in the body.

The miracidium from *P. crucibulum* (Fig. 2) measured 24 × 15 µm (23-25 × 13-15 µm range), around five times shorter than *F. hepatica*. The cilia covered the whole surface of the body, arranged in two-row of epithelial plates, and not in several plates as in *F. hepatica*. A long cilia with 12.7 (11.8-13.7) µm in length. Stylet located outside of the apical gland. Terebratorium and eyespots absent. Four germinal cells and eight nucleus of somatic cells were also observed.

## Discussion

In the Atlantic coast, two mussel species can serve as first intermediate host to *P. crucibulum*, they are *Mytilus edulis* and *M. galloprovincialis* (Matthews, 1973; Cousteau *et al.*, 1990; Teia dos Santos & Coimbra, 1995; McGladdery *et al.*, 1999). Therefore, to study the trematode strategy for reaching the first intermediate host, it is relevant to understand the dynamics of its life cycle. *P. crucibulum* life cycle was studied by Matthews (1973), who also observed different stages of egg development (immature and mature) within the uterus of *P. crucibulum*, what is corroborated by the findings reported in our work. The percentage of each development stage was similar in *P. crucibulum* and *F. hepatica*. However, the minimum number of eggs/worm was different in both species, being higher in *P. crucibulum* than in *F. hepatica*, besides their different adult size (the former are 4-5 times smaller than the later). With regard to the dimensions of the eggs recorded here, we can see that they are similar to the ones recorded in other *Prosorhynchus* species, such as: *P. aculeatus*, *P. maternus*, *P. pacificus*, *P. atlanticus* and *P. australis* (Jones, 1943; Winter, 1960; Etchegoin *et al.*, 2005; Bray & Justine, 2006). However, they are not similar to the ones found in *F. hepatica* that are 4-5 times bigger (Duwel, 1982).

Therefore, we can note that *P. crucibulum* and *F. hepatica* have different strategies of egg production; the former has small eggs, and smaller miracidia, in large number, and the later has large eggs and larger miracidia in small number. This could be related to different strategies to achieve the first intermediate host, the mollusc.

The two strategies of the miracidium that are currently recognized in the literature are: some larvae have an active way of infection, while others have a passive way. According to Galaktionov & Dobrovolskij (2003), these are associated with different morphologies of the larva. *F. hepatica* miracidium, which is an active infecting larva, presents a large size, an apical papilla, several ciliated epithelial plates and eyespots. While, in *P. crucibulum* miracidium we have reported several features that belong to the second group: small size, only two ciliated epithelial plates, terebratorium absent, stylet present and situated outside the apical gland and eyespots absent. In summary, we can say that the morphology of the miracidium from *P. crucibulum* is very simplified compared with that of *F. hepatica*. The same pattern was also recorded for *P. squamatus* and was associated to its passive way of infection (Galaktionov & Dobrovolskij, 2003). The active infection of the first host for bucephalids, generally accepted, was questioned by those authors and is here confirmed by the reported features that they most probably have a passive way of infection.
